# Electroacupuncture Ameliorates Cerebral I/R-Induced Inflammation through DOR-BDNF/TrkB Pathway

**DOI:** 10.1155/2020/3495836

**Published:** 2020-03-16

**Authors:** Yue Geng, Yeting Chen, Wei Sun, Yingmin Gu, Yongjie Zhang, Mei Li, Jiajun Xie, Xuesong Tian

**Affiliations:** ^1^Innovation Research Institute of Traditional Chinese Medicine, Center for Drug Safety Evaluation and Research, Shanghai University of Traditional Chinese Medicine, Shanghai, China; ^2^Department of Physiology, Shanghai University of Traditional Chinese Medicine, Shanghai, China

## Abstract

The beneficial effects of electroacupuncture (EA) at Shuigou (GV26) and Neiguan (PC6) on poststroke rehabilitation are critically related to the activation of the delta-opioid receptor (DOR). The underlying anti-inflammatory mechanisms in DOR activation and EA-mediated neuroprotection in cerebral ischemia/reperfusion (I/R) injury were investigated in the current study. Cell proliferation and apoptosis were detected by morphological changes, cell counting kit-8 (CCK-8) assay, lactate dehydrogenase (LDH) release, and TUNEL staining. The mRNA levels were evaluated by using real-time quantitative polymerase chain reaction (RT-qPCR), and the protein expression was measured by western blot or enzyme-linked immunosorbent assay (ELISA) *in vitro*. Infarct volume was examined by cresyl violet (CV) staining, neurologic recovery was assessed by neurological deficit scores, and pro- and anti-inflammatory cytokines were determined by immunofluorescence *in vivo*. DOR activation greatly ameliorated morphological injury, reduced LDH leakage and apoptosis, and increased cell viability. It reversed the oxygen-glucose deprivation/reoxygenation- (OGD/R-) induced downregulation of DOR mRNA and protein, as well as BDNF protein. DOR activation also reduced proinflammatory cytokine gene expression, including TNF-*α*, IL-1*β*, and IL-6, and at the same time, increased anti-inflammatory cytokines IL-4 and IL-10 in OGD/R challenged PC12 cells. EA significantly reduced middle cerebral artery occlusion/reperfusion- (MCAO/R-) induced infarct volume and attenuated neurologic deficit scores. It markedly increased the expression of IL-10 and decreased IL-1*β*, while sham EA did not have any protective effect in MCAO/R-injured rats. DOR activation plays an important role in neuroprotection against OGD/R injury by inhibiting inflammation via the brain-derived neurotrophic factor/tropomyosin-related kinase B (BDNF/TrkB) pathway. The neuroprotective efficacy of EA at Shuigou (GV26) and Neiguan (PC6) on cerebral I/R injury may be also related to the inhibition of inflammatory response through the DOR-BDNF/TrkB pathway.

## 1. Introduction

Stroke is the third leading cause of mortality in most western countries, preceded only by coronary heart disease and malignant tumor. However, it has been the leading cause of death in China in recent years, threatening people's health and affecting their quality of life [1]. Among various types of stroke, ischemic accounts for about 87% of all strokes [2].

Reported scientific findings have highlighted that inflammatory response plays a crucial role in cerebral ischemia/reperfusion (I/R) injury and aggravates ischemic and anoxic injury, worsening patient prognosis [3]. Thus, inhibiting inflammation after cerebral I/R injury has far-reaching clinical implications. As an effective therapy and complementary medicine, acupuncture has been used in China for centuries. Recently, increasingly clinical and experimental studies have demonstrated that electroacupuncture (EA), an extended technique based on traditional acupuncture and combined with modern electrotherapy, has been proved to be an effective therapeutic method for cerebral ischemia and is related to the anti-inflammatory response [4–7].

*δ*-opioid receptor (DOR) is an oxygen-sensitive protein, which is sensitive to ischemia and hypoxia. The activation of DOR is neuroprotective against cerebral I/R injury through various mechanisms, including maintaining ionic homeostasis against excitotoxic and antioxidative stress [8, 9]. Recently, a large amount of research suggests that the alleviation of inflammatory response, such as downregulation of the expression of proinflammatory mediators TNF-*α*, IL-6, and IL-1*β* and upregulation of anti-inflammatory factors IL-4 and IL-10 [10–12], is also involved in the neuroprotection after cerebral I/R. The binding of brain-derived neurotrophic factor (BDNF) to its high affinity receptor tropomyosin-related kinase B (TrkB) has been recognized as one of the anti-inflammatory pathways [13, 14]. Interestingly, recent evidence also shows that BDNF is probably regulated by DOR in cerebral I/R injury [15–17]. Therefore, it remains to be verified whether or not DOR can regulate the BDNF/TrkB pathway and then alleviate cerebral I/R inflammatory response to protect against the cerebral I/R injury.

Reports suggest that one of the mechanisms of EA protection in cerebral I/R injury appears to be regulating the expression of BDNF and its receptor TrkB [18, 19]. Over the years, studies have shown that the treatment of EA has relieved the symptoms for analgesia and other diseases with activation of the endogenous opioid system in the brain [20–22]. Particularly, recent studies show that EA at Shuigou (GV26) and Neiguan (PC6) has obvious effects for cerebral I/R injury among the many acupuncture points available for treatment, and its therapeutic mechanism may be associated with the activation of DOR, one of the three classic opioid receptor subtypes [23, 24]. Nonetheless, it is unclear whether the DOR-BDNF/TrkB signaling pathway-mediated anti-inflammatory effect is involved in the therapeutic efficacy of EA at Shuigou (GV26) and Neiguan (PC6) on cerebral I/R injury.

Hence, this investigation is primarily aimed to address the following two fundamental issues using a combination of cell oxygen-glucose deprivation/reoxygenation (OGD/R) and rat middle cerebral artery occlusion/reperfusion (MCAO/R) models: (1) activation of DOR attenuates OGD/R-induced inflammation through the BDNF-TrkB signaling pathway; and (2) DOR-BDNF/TrkB signaling pathway is involved in the anti-inflammatory mechanisms of EA at Shuigou (GV26) and Neiguan (PC6) in cerebral I/R injury.

## 2. Materials and Methods

### 2.1. Chemicals and Reagents

Tan67 and Naltrindole (NTI) were purchased from Tocris bioscience (Cat: 0921, 0740). Cresyl violet (CV) was purchased from Sigma-Aldrich (Cat: C5402). Hoechst 33258, BCA assay kit (BSA standard solution) and the protein extraction kit were purchased from Beyotime Biotechnology (Cat: C1017, P0010, and P0013B). Mouse monoclonal anti-IL-10 (A-2) antibody was purchased from Santa Cruz Biotechnology (Cat: SC-365858). Rabbit polyclonal anti-IL-1*β* antibody was obtained from Abcam Inc (Cat: ab9722). Rabbit polyclonal anti-DOR antibody was obtained from Millipore Sigma (Cat: AB1560). Mouse monoclonal anti-*β*-actin antibody was obtained from Sigma-Aldrich (Cat: A5441). Alexa Fluor 647-conjugated Affinipure Donkey Anti-Rabbit IgG (H + L), Alexa Fluor 488-conjugated Affinipure Donkey Anti-Mouse IgG (H + L), Peroxidase AffiniPure Goat Anti-Rabbit IgG (H + L), and Peroxidase AffiniPure Goat Anti-Mouse IgG (H + L) are Jackson Immuno Research products (Cat: 711-605-152, 715-545-150, 111-035-003, and 115-035-003). Laemmli sample buffer is from Bio-rad (Cat: 1610747).

### 2.2. Cell Culture

PC12 cells were purchased from Cell Bank of Type Culture Collection of the Chinese Academy of Science (Shanghai, China) and then cultured in the full culture medium, RPMI-1640 (Gibco, Carlsbad, CA, USA), containing 10% heat-inactivated fetal bovine serum (Gibco) and 1% penicillin/streptomycin (Gibco), in a humidified incubator (5% CO_2_, 95% air) at 37°C. Cells were fed with fresh medium every other day, and the cells of passages 4–6 were used for experiments.

### 2.3. Oxygen-Glucose Deprivation/Reoxygenation and Cell Culture Treatment

Cells of the same passage were randomly divided into 5 groups: (1) control group; (2) OGD/R group; (3) agonist group (OGD/R + Tan67); (4) antagonist group (OGD/R + NTI); (5) coadministration group (OGD/R + NTI + Tan67). The OGD/R in PC12 cells was performed as reported before [25, 26]. In brief, PC12 cells were washed with phosphate-buffered saline (PBS) twice and cultured in glucose-free RPMI-1640 culture media and then placed in a hypoxia chamber (95% N_2_ and 5% CO_2_) for 6 h at 37°C. The cells were then fed back with full culture medium and cultured under normal conditions for additional 24 h. Control cells were incubated in the regular cell culture incubator under normoxic conditions for the same duration. DOR agonist Tan67 or antagonist NTI was dissolved with PBS (pH 7.2) and then filtered (0.22 *μ*m) to obtain stock solutions (25 mM). Immediately before use, the stock solution was diluted in the cell culture medium to 10 *μ*M. In the agonist and antagonist groups, PC12 cells were pretreated with Tan67 or NTI alone for 30 min, while in the coadministration group, NTI incubation starts 30 min before adding Tan67, following by 6 h of OGD and 24 h of reperfusion. The same amount of dissolvent was added into control and OGD/R cultures to guarantee all groups under the same experimental procedure.

### 2.4. Animals

Healthy adult male Sprague-Dawley rats (230 ± 10 g of body weight) were obtained from Shanghai Laboratory Animal Center and maintained under specific pathogen-free conditions in Shanghai University of Traditional Chinese Medicine. Rats were housed in cages under a 12-hour light/dark cycle, 60%–70% relative humidity, and a temperature of 22°C ± 2°C, with free access to water and food. Animals were allowed one week to acclimate prior to experimentation. All procedures were performed in accordance with the Provision and General Recommendation of Chinese Experimental Animals Administration Legislation and approved by the Experimental Animal Ethics Committee of Shanghai University of Traditional Chinese Medicine (PZSHUTCM18121401).

### 2.5. Experimental Groups and Lateral Ventricle Injection

Forty rats were randomly assigned into the following five groups: (1) sham group (Sham), (2) MCAO group (MCAO), (3) sham EA group (MCAO + S + EA), (4) EA group (MCAO + EA), and (5) EA + DOR antagonist group (MCAO + NTI + EA). NTI was freshly dissolved in saline to a final concentration of 100 nM and then filtered (0.22 *μ*m). Ten microliters of NTI (100 nmol) was stereotaxically injected into the right lateral ventricle 30 min before MCAO started (Shanghai Alcott Biotechnology Co., Ltd). Ten *μ*l Hamilton syringe (10 *μ*l, 1701 N) was used to inject at the rate of 1 *μ*l/min. After the injection, the needle was left in place for a few minutes before being retracted slowly and the wound was cleaned and sutured. The rat in the other group received the corresponding volume of saline. The coordinates of the injection site were 0.2 mm posterior to the bregma, 1.4 mm lateral, and 4 mm below the skull surface, with a flat skull position.

### 2.6. MCAO/R Model

The MCAO/R model is the most widely used animal model to mimic the cerebral I/R injury [27]. The MCAO/R (90 min/24 h) procedure was performed according to the methods of Longa with minor modifications [28]. In brief, the rats were anesthetized with pentobarbital (50 mg/kg, i.p.), and the right common carotid artery (CCA), external carotid artery (ECA), and internal carotid artery (ICA) were gently exposed through a ventral midline incision. A 3-0 monofilament nylon suture (Dermaron, Davis-Geck, USA) with a rounded tip was inserted into the right ICA though the ECA, with a depth of approximately 18 ± 2 mm. The suture was carefully removed 90 min after the ischemia for 24 h of reperfusion. Sham-operated rats were subjected to the same procedures described above without suture insertion. The rectal temperature was monitored and maintained at 37 ± 0.5°C throughout the experiment.

### 2.7. EA Treatments

At 30 minutes of ischemia, rats in the MCAO + EA and MCAO + NTI + EA group underwent EA stimulation for 90 min at Shuigou (GV26) and the left side of Neiguan (PC6). Acupoints of Shuigou (GV26) and Neiguan (PC6) were selected according to the “animal acupuncture points map” reported by the Experimental Acupuncture–Moxibustion Research Association of China (Academy of Acupuncture–Moxibustion). The acupoints were stimulated with an intensity of 1-2 mA and a disperse-dense frequency of 5/20 Hz (adjusted to the muscle twitch threshold) by using the EA instrument (SDZ-V EA, Suzhou, China). In the sham EA group, acupuncture needles were inserted superficially into the same acupoints without electrical stimulation [24].

### 2.8. Cell Morphology and Viability

To evaluate changes in cell morphology, the PC12 cells were grown in 6-well plates and treated as described above, and cell morphology was observed under an inverted microscope (Nikon, Japan).

#### 2.8.1. Cell Viability

Cell viability was determined by the cell counting kit-8 (CCK-8) assay and lactate dehydrogenase (LDH) release. CCK-8 assay was performed according to the manufacturer's instructions (Beyotime Biotechnology, Shanghai, China) and similar to our previous study [29]. In brief, PC12 cells were seeded in 96-well plate at 1 × 10^5^ cells/mL and allowed to adhere properly for 24 h. After OGD/R insult, 10 *μ*l of the CCK-8 solution was added to each well and was incubated for another 1.5 h under the same incubator conditions. Subsequently, the optical density (OD) value of each well at the absorbance 450 nm was determined using a multiwell microplate reader (Synergy HT, BioTek). The mean OD of all wells in the indicated groups was used to calculate the percentage of cell viability as follows: percentage of cell viability = (*A*_treatment_ − *A*_blank_)/(*A*_control_ − *A*_blank_) × 100% (where*A* = absorbance). LDH release was evaluated using a commercially available kit (A020-2-2, Jiancheng, Nanjing, China). In brief, after removal of the culture supernatants from each well, 1% Triton X-100 lysis solution was added to the wells. Cells were placed in the lysis buffer for 30 min at 37°C. Then, the collected cell culture supernatants and lysates were centrifuged at 3000*g* for 15 min, respectively. Subsequently, the centrifuged supernatant was transferred to a new 96-well plate for LDH activity analysis according to manufacturer's instructions. The absorbance was determined at 450 nm with a microplate reader (Synergy HT, BioTek). Background absorbance from the cell-free buffer solution was subtracted from all absorbance measurements. The percentage of LDH released to cell culture medium in total LDH was calculated: LDH leakage = LDH in cell culture supernatant/(LDH in cell culture supernatant + LDH in cell lysate) × 100%.

#### 2.8.2. TUNEL Staining

The apoptotic cells were stained with TUNEL reagents according to the manufacturer's instructions (Roche Applied Science, Rotkreuz, Switzerland). After OGD/R, the cells were washed with PBS three times and then fixed in 4% paraformaldehyde at 37°C for 20 min. The cells were then rinsed with PBS three times and incubated in a permeabilizing solution (0.1% Triton X-100 in sodium citrate) for 3 minutes. After rinsing in PBS, the cells were incubated in TUNEL with an enzyme-to-label ratio of 1 : 9 at 37°C for 1 h and nuclei staining with Hoechst 33258 working solution (5 mg/mL) for 10 min at room temperature in the dark. After washed with PBS three times, the immunofluorescent images were observed by using a Laser Scanning Confocal Microscope (TCS SP8, Leica, Germany). TUNEL positive PC12 cells were counted in 5 randomly selected high-power fields (HPF, 400x magnification) per culture dish, respectively, and expressed as the number of apoptotic cells in each HPF.

### 2.9. RNA Isolation, cDNA Synthesis, and Polymerase Chain Reaction

The mRNA levels of DOR, TNF-*α*, IL-1*β*, IL-6, IL-4, and IL-10, in PC12 cells were measured by real-time quantitative polymerase chain reaction (RT-qPCR). The total RNA was isolated using Trizol reagent (Invitrogen, Paisley, UK). Subsequently, 800 ng RNA was reversely transcribed into cDNA at 37°C for 15 min followed by 85°C for 5 sec using TB Green™ Premix Ex Taq™ (Takara, Shiga, Japan). RT-qPCR was performed with the ABI 7500 PCR system (ABI, USA). The PCR cycling conditions were 40 amplification cycles of denaturation at 95°C for 30 s, followed by 94°C for 5 sec and 60°C for 34 sec. The quantified value of each sample was normalized to *β*-actin expression in the same sample, which was amplified simultaneously with the target genes. The relative gene expression was quantified using the 2^−∆∆*t*^ method. Each sample was tested in triplicate. Primers for DOR, TNF-*α*, IL-1*β*, IL-6, IL-4, and IL-10 were synthesized by Kingsray Biotechnology (Nanjing, China). The sequences of the primers used in this study are listed in Table 1.

### 2.10. Western Blot

Western blot was used to determine DOR protein expression as previously described [30]. In short, following OGD/R, the cells were rinsed twice with ice-cold PBS and total protein was extracted using RIPA buffer (P0013B, Beyotime). Protein concentration was then measured by BCA protein assay (P0010, Beyotime). Equal amount of protein samples (20 *μ*g/10 *μ*L) were boiled at 100°C in Laemmli sample buffer (Bio-Rad Laboratories, Inc., USA) for 5 min and then loaded and electrophoresed on 10% SDS-polyacrylamide gel (product information). Proteins were transferred from gel to 0.22 *μ*m nitrocellulose (NC) membrane (Millipore, Billerica, MA, USA) using a wet transfer system (Bio-Rad). The membranes were blocked with 5% nonfat milk in TBS containing 0.1% Tween-20 for 1 h at room temperature and then incubated with primary antibodies (1 : 3000 rabbit anti-DOR, and 1 : 10,000 mouse *β*-actin antibody) at 4°C overnight. The membranes were washed three times for 10 min each with TBS containing 0.1% Tween-20 and incubated with the corresponding horseradish peroxide-conjugated secondary antibodies (1 : 5000 goat anti-rabbit IgG and 1 : 5000 goat anti-mouse IgG) at room temperature for 2 h. Next, membranes were washed three times with 0.1% TBST, and the immune-reactive bands were detected using enhanced chemiluminescence reagent (Beyotime) and scanned with the ChemiDoc XRS imaging system (Bio-Rad). Gray-scale analysis was analyzed by Image-Pro Plus 6.0 analysis system (Media Cybernetics Inc., USA), and the protein expression was normalized to the expression of *β*-actin.

### 2.11. Measurement of BDNF Protein Levels

BDNF levels in the cell culture supernatants and cell lysates were measured by BDNF enzyme-linked immunosorbent assay (ELISA) (R&D Systems, DBNT00, Minneapolis, USA). In brief, the culture supernatants and cell lysate in 1% Triton X-100 lysing solution were collected and then centrifuged at 3000*g* for 15 min, respectively. Subsequently, BDNF content was determined in accordance with the manufacturer's instructions. Absorbance was measured at 450 nm with a microplate reader (Synergy HT, BioTek). The BDNF protein levels were calculated in comparison with the standard curve.

### 2.12. Scoring of Neurological Deficits

Neurologic deficit scores were evaluated at 24 h of reperfusion based on the method of Longa et al. [28]. The scores for the neurological behavioral were as follows: 0 = no deficit, 1 = failure to extend left forepaw fully, 2 = circling to the left, 3 = falling to the left, and 4 = no spontaneous walking with a depressed level of consciousness. The scores were rated double-blind.

### 2.13. Free Floating Coronal Section Preparation

At 24 h of reperfusion, under deep anesthesia with sodium pentobarbital (80 mg/kg, i.p.), the brains were carefully removed after transcardial perfusion with 0.9% saline solution followed by 4% ice-cold phosphate-buffered paraformaldehyde (PFA). Subsequently, the rat brains were fixed in 4% PFA for 12 h and then immersed sequentially in 20% and 30% sucrose solutions in 0.1 M phosphate buffer (pH 7.4) until they sank. Finally, brains were cut into 30 *μ*m coronal sections from bregma 1.60 to −4.80 mm on a freezing microtome (CM1950, Leica, Germany) and stored at −20°C in cryoprotectant solution.

### 2.14. Infarct Volume Assessment

Sections at 1.60 to −4.80 mm from bregma were used for CV staining. The volume of the brain infarct was measured in each slice at 360 *μ*m intervals with Image-Pro Plus 6.0 analysis system (Media Cybernetics Inc.). Data were presented as the percentage in the volume of the entire brain.

### 2.15. Immunofluorescence

Sections at 1.0 to 0.48 mm from bregma were used for immunofluorescence staining as previously described [15]. Free-floating sections were washed in 0.01 M PBS three times (5 min each). Sections were then incubated with 0.3% H_2_O_2_ for 30 min and placed in blocking buffer containing 10% donkey serum and 0.3% Triton X-100 in 0.01 M PBS for 30 min at room temperature. Subsequently, the sections were incubated with primary antibodies against rabbit polyclonal IL-1*β* (1 : 100) or mouse monoclonal anti-IL10 (1 : 50) overnight at 4°C, respectively. After washing with PBS, the sections were incubated with corresponding secondary antibodies (1 : 500) for 1 h at 37°C. Nuclei were counterstained with Hoechst 33258 (5 *μ*g/mL) for 10 min in the dark. Finally, following additional three washes in PBS, these sections were mounted on glass slides and cover slipped using fluorescent mounting media. Images were captured using a confocal laser scanning microscope (TCS SP8, Leica, Germany) with 630x magnification at excitation 490 nm (Alexa Fluor 488), 640 nm (Alexa Fluor 647), and 360 nm (Hoechst 33258). Negative control sections received the identical process without primary antibodies and showed no specific staining.

Double-positive for IL-1*β* and Hoechst 33258 cells were counted in 10 randomly selected fields (630x magnification) using an Image-Pro Plus 6.0 analysis system (Media Cybernetics Inc.), and the average percentage of double-positive cells was presented as double-positive/total cells × 100%. IL-10 positive staining was determined according to the mean and integrated optical density (IOD). In brief, 5 random HPFs (630x magnification) were selected in each immunofluorescence slice. Quantitative analysis of total of IL-10 positive area and IOD of positive staining area was performed according to the software as described above. The mean density = IOD SUM/area SUM.

### 2.16. Statistical Analyses

Statistical analysis was performed using the SPSS software (version 23; IBM Corp., Armonk, NY, USA). Data are presented as the mean ± standard error of mean (SEM). When equal variance was assumed, the data were analyzed using one-way analysis of variance (ANOVA) followed by Dunnett's post hoc test for multiple comparisons. When equal variance was not assumed, the data were compared using the nonparametric test. To avoid false positives caused by multiple comparisons, the Bonferroni's correction was performed to adjust the test level (0.05/n). The neurologic deficit scores were expressed as the median (range) and were analyzed with a nonparametric method (Kruskal–Wallis test) followed by the Mann–Whitney *U* test with Bonferroni's correction. Probability values of *P* < 0.05, *P* < 0.01, and *P* < 0.001 were considered to indicate a statistically significant difference.

## 3. Results

### 3.1. DOR Activation Reduced Cellular Injury Induced by OGD/R in PC12 Cells

**Figure 1:**
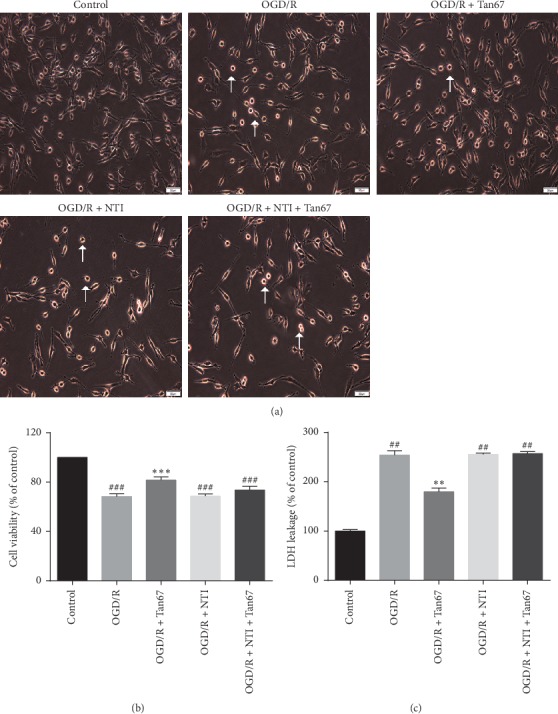
DOR activation increases cell viability in OGD/R-exposed PC12 cells. The cellular morphological changes, cell viability, and LDH level were evaluated after exposure to 6 h OGD followed by 24 h reperfusion (OGD/R). The DOR agonist or antagonist treatment is 30 min before OGD. In the coadministration group, antagonist NTI was added to the cell culture medium 30 min before the agonist Tan67 incubation. (a) Representative images of PC12 cells in the sham group, OGD/R group, agonist group, antagonist group, and coadministration group. Cell viability was measured by (b) CCK-8 assay and (c) LDH release. Activation of DOR attenuated the PC12 cells injury, whereas it was totally abolished by preincubation of DOR antagonist. Scale bar = 50 *μ*m. *N* = 6 in each group. ^###^*P* < 0.001 and ^##^*P* < 0.01 vs. control group. ^*∗∗∗*^*P* < 0.001 and ^*∗∗*^*P* < 0.01 vs. OGD/R group.

The PC12 cells line, which derives from rat adrenal medulla tumor, has been widely used for cell signaling studies [31]. The positive findings of this cell line can be validated in primary neuronal cultures and could also be adapted for OGD/R research [32, 33]. To examine the neuroprotective effect of the DOR on OGD/R-induced cell injuries, the cellular morphology, cell viability, and LDH release were assessed immediately after the OGD/R by inverted microscope, CCK-8 assay, and LDH leakage assay. As shown in Figure 1(a), the morphological characteristics of the PC12 cells in the control group included large cell amount, strong refractive index, and vigorous axonal growth; moreover, the synapses were clearly interwoven into a network. The OGD/R exposure resulted in a reduction in cell number, and the morphology exhibited round, slender, and degenerated cell debris (Figure 1(a)). The number of cells increased drastically, and normal morphology was restored if PC12 was pretreated with the DOR agonist Tan67 (10 *μ*M) before OGD/R exposure, as evidenced by observations under microscope (Figure 1(a)). However, the morphology and numbers of NTI (10 *μ*M) and NTI + Tan67 pretreated cells were similar to that in the OGD/R group (Figure 1(a)). The results were further quantified by the CCK-8 assay, which measures cell viability, and were expressed as % cell viability of control (Figure 1(b)). The cell viability markedly decreased by OGD/R exposure (68.3 ± 2.2%, *P* < 0.001) (Figure 1(b)). A statistically significant increase in cell viability was detected in cells preincubated with the DOR agonist Tan67 (81.7 ± 2.6%, *P* < 0.001) (Figure 1(b)). However, the protective effect of Tan67 was blocked by concomitant incubation with the DOR antagonist NTI (73.7 ± 3.0%, *P* > 0.05) (Figure 1(b)), suggesting that DOR activation contributes to the protection against OGD/R-induced cell death. The application of NTI itself did not change cell viability (68.7 ± 1.8%, *P* > 0.05) in the OGD/R-treated cells (Figure 1(b)).

Next, to further confirm the effect of DOR on OGD/R-induced PC12 cell injury, LDH leakage assay was performed (Figure 1(c)). The increased release of LDH is recognized as a reliable index of neuronal injury [8]. The LDH leakage rate was significantly increased after PC12 cells were exposed to OGD/R (254.2 ± 8.8%, *P* < 0.01), comparing with the control group (100.0 ± 3.1%) (Figure 1(c)). However, activating DOR with Tan67 (10 *μ*M) clearly attenuated the LDH leakage rate (179.7 ± 7.7%, *P* < 0.01) in comparison to the OGD/R group (Figure 1(c)), whereas, as shown in the last bar in Figure 1(c), the increase in LDH leakage in the coadministration group (257.3 ± 3.7%, *P* > 0.05) was the same as that in none drug-treated OGD/R group, indicating that the Tan67-mediated neuroprotection was abolished by the blockade on DOR activation. NTI alone did not make any difference in the LDH release (255.4 ± 2.7%, *P* > 0.05) compared with the OGD/R group (Figure 1(c)). These data indicate that DOR plays a significant role in neuroprotection against OGD/R-induced injury.

### 3.2. Effect of DOR Activation on Cell Apoptosis

**Figure 2:**
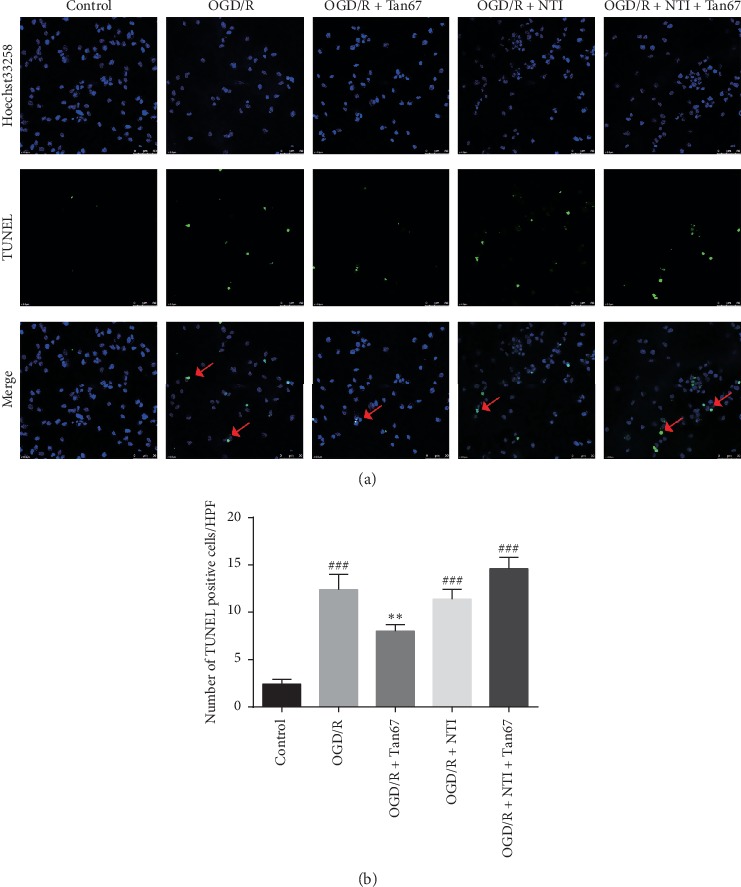
DOR activation protects against OGD/R-induced cell apoptosis. The apoptotic cells were evaluated after exposure to 6 h OGD followed by 24 h reperfusion (OGD/R). (a) The top panels are representative images of TUNEL staining (400x) in indicated groups. Red arrows indicate colocalization (yellow) between TUNEL-positive apoptotic cells (green) and cell nuclei (blue) in PC12 cells. Cell nuclei were visualized by Hoechst 33258 staining (blue). (b) The bottom panel is the statistical analysis of the number of apoptotic cells. DOR activation reduced cell apoptosis, and NTI blocked the effect. Scale bar = 50 *μ*m. *N* = 3 in each group. ^###^*P* < 0.001 vs. control group and ^*∗∗*^*P* < 0.01 vs. OGD/R group.

To evaluate the effects of DOR activation on OGD/R-induced apoptosis in PC12 cells, the TUNEL assay was applied. As shown in Figure 2(a), apoptosis was identified by the nuclear staining in green. TUNEL and Hoechst 33258 double-positive cells (yellow) in each group were counted manually by two independent observers in 6 random microscopic fields, and the numbers of double-positive cells were subsequently calculated (Figure 2(b)).

There were almost no apoptotic changes noted in the nuclei in the control group (Figure 2(b)). Extensively TUNEL-positive apoptotic cells were observed in PC12 culture exposed to OGD/R (12.4 ± 1.6, *P* < 0.001), while pretreatment with Tan67 remarkably decreased the number of TUNEL-positive cells (8.0 ± 0.7, *P* < 0.01) (Figure 2(b)). However, pretreatment with NTI (14.6 ± 1.2, *P* > 0.05) attenuated the beneficial effect of Tan67, suggesting that DOR activation might inhibit apoptosis (Figure 2(b)). NTI alone did not exert a significant effect on OGD/R-induced apoptosis (11.4 ± 1.0, *P* > 0.05) (Figure 2(b)). This result indicates that DOR activation may inhibit apoptosis.

### 3.3. Effect of DOR Activation on the Production of Proinflammatory Cytokines (TNF-a, IL-1*β*, and IL-6) and Anti-Inflammatory Cytokines (IL-4 and IL-10)

**Figure 3:**
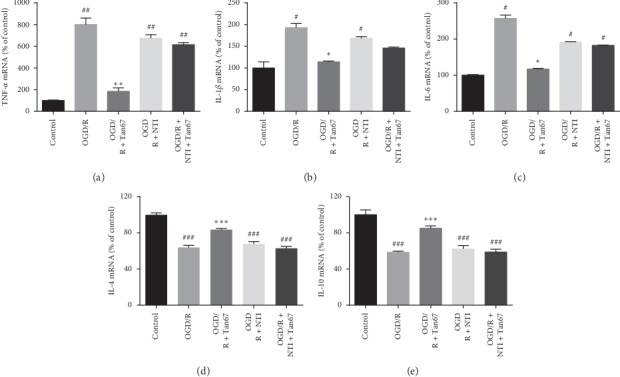
DOR activation reduces inflammatory response. The expression of proinflammatory cytokines (e.g., TNF-*α*, IL-1*β*, and IL-6) and anti-inflammatory cytokines (e.g., IL-4 and IL-10) was evaluated by RT-qPCR at 24 h of reperfusion following 6 h OGD. Statistical analysis of (a) TNF-*α*, (b) IL-1*β*, (c) IL-6, (d) IL-4, and (e) IL-10 mRNA expression is shown. DOR activation prevented the OGD/R-induced upregulation of proinflammatory factors (TNF-*α*, IL-1*β*, and IL-6) and downregulation of the anti-inflammatory factors (IL-4 and IL-10), and the effects were blocked by NTI treatment. *N* = 3 in each group. ^#^*P* < 0.05 and ^###^*P* < 0.001 vs. control group. ^*∗*^*P* < 0.05, ^*∗∗*^*P* < 0.01, and ^*∗∗∗*^*P* < 0.001 vs. OGD/R group.

Inflammation response plays a pivotal role in the pathological process of cerebral I/R injury. Further to clarify the specific mechanism of DOR activation in neuroprotection, the RT-qPCR was performed to evaluate how DOR affects the expression of inflammation-related factors. The mRNA levels of TNF-*α*, IL-1*β*, and IL-6 in the OGD/R group (800.4 ± 60.1%, 192.9 ± 9.6%, and 257.2 ± 9.2%, *P* < 0.01 or *P* < 0.05) (Figures 3(a)–3(c)) were dramatically increased compared with the control group. In contrast, the mRNA levels of IL-4 and IL-10 were decreased after OGD/R (63.8 ± 2.4% and 58.6 ± 1.1%, *P* < 0.001) (Figures 3(d) and 3(e)). Pretreatment with Tan67 dramatically decreased TNF-*α*, IL-1*β*, and IL-6 expression (185.0 ± 31.9%, 114.3 ± 1.8%, and 117.2 ± 2.0%, *P* < 0.01 or *P* < 0.05) (Figures 3(a)–3(c)) and increased IL-4 and IL-10 expression (83.4 ± 1.5%, 85.1 ± 2.8%, *P* < 0.001) (Figures 3(d) and 3(e)) in PC12 cells compared with the OGD/R group. However, pretreatment with NTI reversed the protective effect of Tan67, while NTI application alone had no effect on the mRNA levels, as evidenced by the expression of TNF-*α*, IL-1*β*, IL-6, IL-4, and IL-10 in the OGD/R + NTI + Tan67 group (615.0 ± 18.8%, 145.9 ± 2.5%, 182.3 ± 1.1%, 63.0 ± 2.1%, and 58.8 ± 3.2%, *P* > 0.05) and the OGD/R + NTI group (673.5 ± 33.8%, 168.5 ± 4.2%, 191.2 ± 1.7%, 67.7 ± 2.8%, and 62.3 ± 3.8%, *P* > 0.05) (Figures 3(a)–3(e)). These results demonstrate that DOR activation reduces proinflammatory cytokines and increases anti-inflammatory cytokines gene expression, thus attenuating OGD/R injury.

### 3.4. Effect of DOR Activation on DOR and BDNF Expression

**Figure 4:**
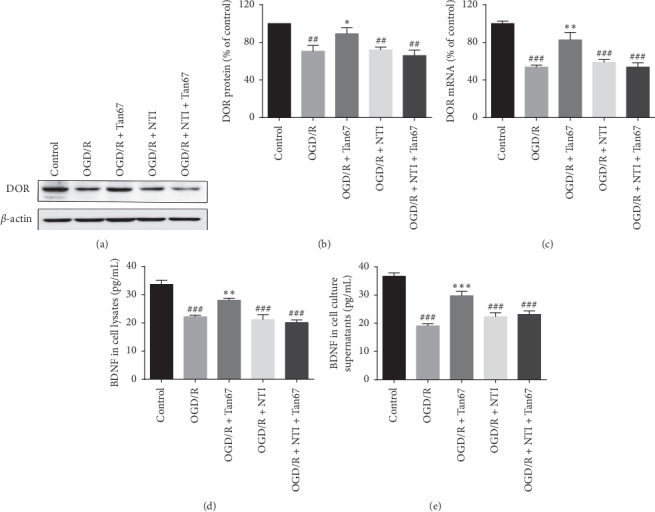
DOR activation elevates DOR and BDNF expression. The mRNA level of DOR was determined by RT-qPCR, and protein expression of DOR and BDNF was measured by western blot or ELISA at 24 h of reperfusion following 6 h OGD. (a) Representative western blot images of DOR expression. (b) Statistical analysis of DOR protein expression. (c) Statistical analysis of DOR mRNA expression. (d) Statistical analysis of BDNF protein expression in cell lysates. (e) Statistical analysis of BDNF protein expression in cell culture supernatants. DOR activation inhibited the OGD-induced decline of DOR mRNA, DOR protein, and BDNF protein expression in PC12 cells, while NTI blocked the effect. *N* = 3 in each group. ^#^*P* < 0.05 and ^###^*P* < 0.001 vs. control group. ^*∗*^*P* < 0.05, ^*∗∗*^*P* < 0.01, and ^*∗∗∗*^*P* < 0.001 vs. OGD/R group.

Next, to study how DOR activation suppresses inflammation in OGD/R exposed PC12 cells, the BDNF signaling pathway was investigated. We determined the expression of DOR and BDNF by western blot, RT-qPCR, and ELISA, respectively. Comparing with control, the expression levels of DOR mRNA (53.8 ± 2.1%, *P* < 0.001) and protein (72.5 ± 5.2%, *P* < 0.01) were significantly reduced in the OGD/R group (Figures 4(a)–4(c)). Pretreatment of PC12 with Tan67 could obviously inhibit the decline of DOR mRNA (82.6 ± 8.1%,*P* < 0.01) and protein (90.7 ± 5.9%, *P* < 0.05) expression induced by OGD/R (Figures 4(a)–4(c)). However, the effect of Tan67 was reversed by pretreatment with NTI (53.7 ± 4.6%, *P* > 0.05; 66.1 ± 5.7%, *P* > 0.05) and had no significant difference compared with the OGD/R group (Figures 4(a)–4(c)). The expression levels of DOR mRNA and protein in the OGD/R + NTI group (59.0 ± 2.8%, *P* > 0.05; 72.1 ± 3.1%, *P* > 0.05) have no notable difference in comparison to the OGD/R group (Figures 4(a)–4(c)) as well.

Consistent with the expression of DOR, similar results were shown on BDNF. BDNF protein level was detected in lysates (33.7 ± 1.5 pg/mL) and cell culture supernatants (36.7 ± 1.2 pg/mL) in the control group (Figures 4(d) and 4(e)). OGD/R exposure markedly reduced BDNF expression both in lysates (22.1 ± 0.5 pg/mL, *P* < 0.001) and cell culture supernatants (19.1 ± 0.7 pg/mL, *P* < 0.001) (Figures 4(d) and 4(e)), while pretreatment with Tan67 resulted in a noticeable enhancement of BDNF in cell lysates (28.0 ± 0.7 pg/mL, *P* < 0.001) and supernatant (29.8 ± 1.5 pg/mL, *P* < 0.001). The coadministration of NTI and Tan67 significantly reversed the effect of Tan67 in lysates (21.1 ± 1.8 pg/mL, *P* > 0.05) and cell culture supernatants (22.4 ± 1.4 pg/mL,*P* > 0.05) (Figures 4(d) and 4(e)). There was no significant difference in the expression of BDNF in lysates (20.1 ± 1.6 pg/mL,*P* > 0.05) and cell culture supernatants (23.2 ± 1.3 pg/mL, *P* > 0.05) in the OGD/R + NTI group compared with the OGD/R group (Figures 4(d) and 4(e)). These results further indicate that the expression of DOR and BDNF was restored by DOR activation.

### 3.5. DOR Antagonist Reversed EA-Induced Neuroprotective Effects in Infarct Volume and Neurologic Deficit Scores

**Figure 5:**
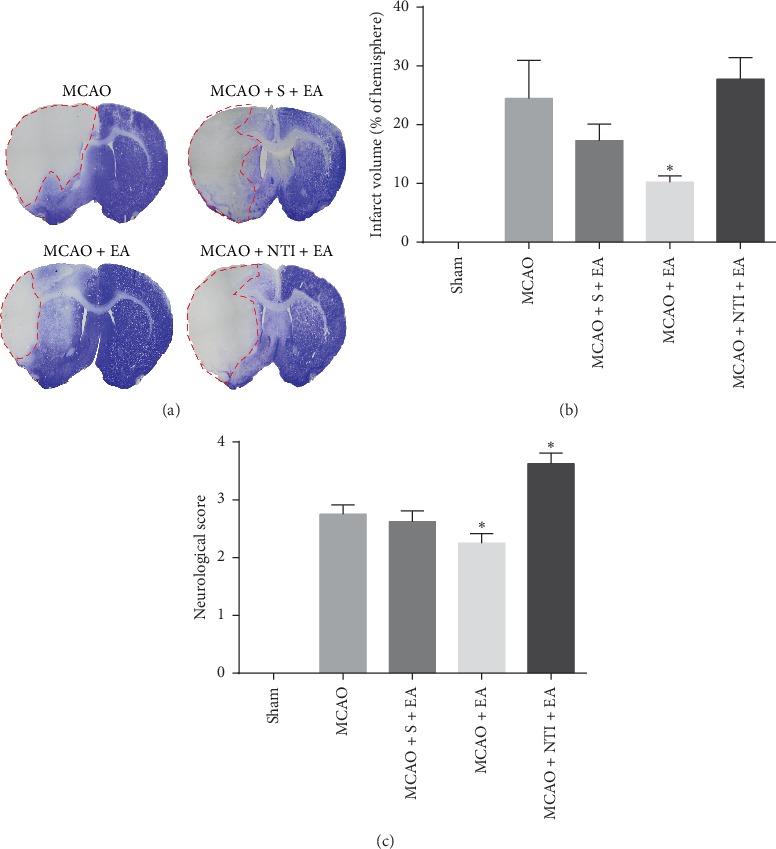
EA-mediated neuroprotective effects in infarct volume and neurologic deficit scores are reversed by DOR antagonist. Infarct volume and neurologic deficit scores were evaluated at 24 h of reperfusion following 90 min MCAO. (a) Representative images of brain infarct volume presented typically in the striatum and cortex indicated by CV staining. The area of pallor, which is inside the red circle, delineates the ischemic core. (b) Statistical analysis of the infarct volume in each group. (c) Statistical analysis of the neurological deficits scores in each group. DOR antagonist NTI abolished the reduction on infarct volume and neurologic deficit scores by EA stimulation at Shuigou (GV26) and Neiguan (PC6). *N* = 8 in each group. ^*∗*^*P* < 0.05 vs. MCAO group.

To investigate whether DOR activation is involved in the neuroprotective effects of EA against cerebral I/R, the infarct volume and neurological deficit scores were evaluated at 24 h of reperfusion (Figure 5). No visible infarct volume and neurological deficit were observed in the sham group (Figures 5(b) and 5(c)). EA treatment at Shuigou (GV26) and Neiguan (PC6) significantly reduced the infarct volume (10.2 ± 1.0%, *P* < 0.05) and neurologic deficit scores (2.3 ± 0.2, *P* < 0.05) compared with the MCAO group (24.5 ± 6.5%; 2.8 ± 0.2) (Figures 5(a)–5(c)). However, NTI administration before MCAO injury reversed the neuroprotective effects of EA on infarct volume (33.7 ± 3.7%, *P* > 0.05) and neurologic deficit scores (3.6 ± 0.2, *P* < 0.05) (Figures 5(a)–5(c)). Sham-operated EA showed no significant improvement compared to MCAO rats in infarct volume (17.3 ± 2.8%, *P* > 0.05) and neurologic deficit scores (2.6 ± 0.2, *P* > 0.05) (Figures 5(a)–5(c)). The results suggest that EA stimulation at Shuigou (GV26) and Neiguan (PC6) may activate DOR and therefore protects against MCAO/R-induced brain injury.

### 3.6. DOR Antagonist Reversed EA-Induced Neuroprotective Effects in Inflammatory Responses

Ample experimental data showed that IL-1*β*, one of the most extensive proinflammatory cytokines, plays a critical role in cerebral I/R injury [34]. At the same time, IL-10, a well-known anti-inflammatory cytokine, was reported to attenuate cerebral I/R injury because of its ability to suppress the production of a variety of proinflammatory cytokines, including IL-1*β*, IL-6, IL-8, and TNF-*α* [35, 36]. Therefore, the expression of IL-1*β* and IL-10 in the cerebral I/R region was detected by the immunofluorescence staining.

**Figure 6:**
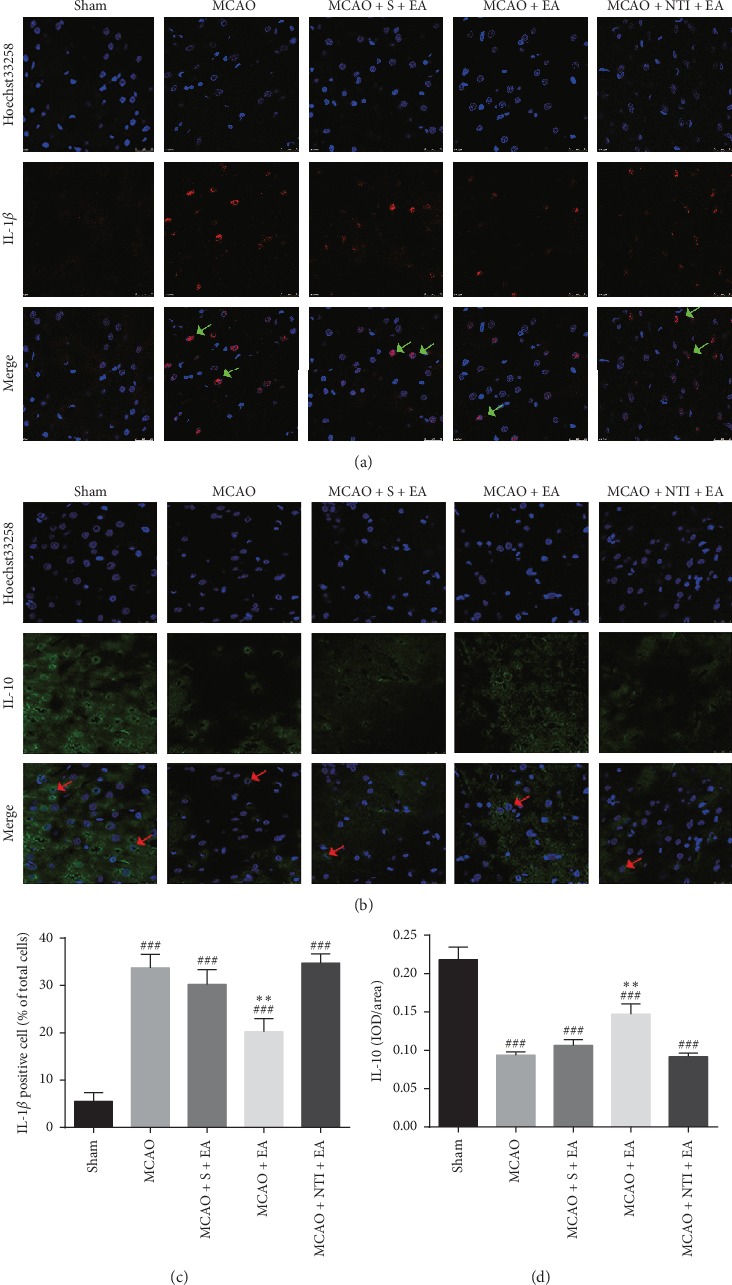
DOR antagonist reversed EA-induced neuroprotective effects in inflammatory responses. Proinflammatory cytokines (IL-1*β*) and anti-inflammatory cytokines (IL-10) were evaluated at 24 h of reperfusion following 90 min MCAO. (a) Representative immunofluorescent staining images of IL-1*β* positive cells in the cortex. (b) Representative images of IL-10 positive immunofluorescent staining in the cortex. (c) Statistical analysis of the percentage of IL-1*β* positive cells. (d) Statistical analysis of the mean density of IL-10 positive staining. Green arrows indicate colocalization (pink) between IL-1*β* positive cells (red) and cell nuclei (blue). Red arrows indicate that IL-10 positive staining (green) is found diffusely distributed across the cytosol and located around the nuclei (blue). Cell nuclei were visualized by Hoechst 33258 staining (blue). EA at Shuigou (GV26) and Neiguan (PC6) reduces the inflammatory factor IL-1*β* and increases the anti-inflammatory factor IL-10, and NTI reversed the EA effect. Scale bar = 50 *μ*m. *N* = 4 in each group. ^###^*P* < 0.001 vs. sham group. ^*∗∗*^*P* < 0.01 vs. MCAO group.

As shown in Figures 6(a) and 6(c), the number of IL-1*β* positive cells in the MCAO group (34.0 ± 2.8%, *P* < 0.001) was higher than that in sham group (5.8 ± 1.9%). Comparable to IL-1*β*, the IL-10 was significantly lower in the MCAO group (0.09 ± 0.00, *P* < 0.001) than in the sham group (0.22 ± 0.02) (Figures 6(b) and 6(d)), as calculated by IOD measurement. Treatment at Shuigou (GV26) and Neiguan (PC6) with EA decreased the number of IL-1*β* positive cells (20.6 ± 2.7%, *P* < 0.01), as well as increased the levels of IL-10 (0.15 ± 0.01, *P* < 0.01) in the cerebral ischemia area (Figures 6(a)–6(d)). Moreover, the lateral ventricle injection of DOR antagonist NTI 30 min before MCAO prevented the changes and brought back the expression of IL-1*β* (35.1 ± 1.9%, *P* > 0.05) and IL-10 (0.09 ± 0.00, *P* > 0.05) to the similar levels as in the MCAO group (Figures 6(a)–6(d)). Sham-operated EA showed no significant improvement compared to MCAO rats on IL-1*β* (30.4 ± 3.0%, *P* > 0.05) and IL-10 (0.11 ± 0.01, *P* > 0.05) (Figures 6(a)–6(d)). These data indicate that DOR activation is likely involved in EA-mediated neuroprotection through suppressing inflammatory responses in cerebral I/R injury.

## 4. Discussion

The principle observations in the current study are as follows: (1) activation of DOR prevents the cell injury induced by OGD/R in PC12 cells; (2) DOR activation markedly reduces OGD/R-induced inflammation; (3) DOR activation restores the mRNA and protein expression of DOR and protein level of BDNF, which were lowered by OGD/R exposure; and (4) EA at Shuigou (GV26) and Neiguan (PC6) suppresses inflammatory responses, reduces ischemic infarct volume, and attenuates neurologic deficit scores in rats with cerebral I/R injury. Together with our previous work [15, 16, 30], we have the following conclusion that DOR-mediated anti-inflammation of BDNF/TrkB pathway plays an important role in ameliorating OGD/R-induced PC12 cells injury, and the beneficial effects of EA treatment may be related to the activation of DOR-BDNF/TrkB pathway in inhibiting the inflammatory response after cerebral I/R-induced neuronal injury.

Four independent assays to detect OGD/R-induced PC12 cells injury (morphological pathology, cell viability, membrane integrity, and DNA breakdown) showed that DOR activation largely attenuated the PC12 cells injury induced by OGD/R, as indicated by reduced morphological changes, increased cell viability, as well as decreased LDH leakage and apoptosis. DOR antagonist totally abolished the DOR activation mediated protective effect, indicating a critical role of the DOR in neuronal protection. This result is supported by previous studies, which showed that DOR activation has potent neuroprotective effect on primary cultured neuron against hypoxia-induced injury [37, 38].

DOR is an oxygen-sensitive protein, and the expression depends on the duration and extent of hypoxia or ischemia. DOR expression is upregulated by short-term or mild hypoxia exposure, which is also called hypoxic preconditioning, and downregulated after prolonged and severe hypoxia/ischemia exposure [8, 30, 39]. Compared treatment with hypoxic preconditioning by exposure to 5% oxygen for 6 h, OGD 6 h followed by reperfusion 24 h is severe hypoxia. Our study showed similar results that both DOR mRNA and protein levels declined in a major way after OGD/R. The DOR agonist Tan67 reverses the downregulation of DOR mRNA and proteins normally produced by OGD/R, whereas the DOR antagonist NTI blocks such protection, suggesting that the Tan67-regulated expression of DOR mainly occurs at the transcriptional and posttranscriptional levels. However, details remain unclear and further experiments need to be carried out.

BDNF binding to its specific receptor-TrkB plays an important role in inhibiting inflammatory response and protecting neurons in both in vitro and in vivo studies. In *in vitro* and *in vivo* models of depression, BDNF was shown to improve depressive-like behavior by upregulating anti-inflammatory cytokine IL-10, IL-4, and TGF-*β*1 and downregulating the pro-inflammatory cytokine IL-1*β*, IL-17, and TNF-*α* [40]. Exogenous BDNF suppressed the expression of proinflammatory factors, including TNF-*α*, IL-1*β*, and IL-6, and increased the expression of the anti-inflammatory factor IL-10 [13]. Evidence from recent studies showed that the expression of BDNF is probably regulated by DOR. For instance, DOR activation significantly upregulated the mRNA expression of BDNF in the frontal cortex through a DOR-mediated mechanism because this effect was blocked by specific DOR antagonist NTI, but not by *μ*- or k-opioid receptor antagonists [17, 41]. Indeed, we previously found that BDNF was colocalized with DOR in DOR-rich regions in the brain [15]. Our previous studies have also demonstrated that DOR activation upregulates BDNF-TrkB signals thereby decreasing the level of TNF-*α* and protecting the cortex against hypoxic injury [16]. In the present study, we observed that the activation of DOR promoted the expression of BDNF in cell lysates and cell culture supernatants, reduced the mRNA levels of TNF-*α*, IL-1*β*, and IL-6, and increased IL-4 and IL-10, further confirming that DOR mediates the anti-inflammatory effect of the BDNF/TrkB pathway.

According to the theory of traditional Chinese medicine, there are many acupoints used in the treatment of stroke. The evidence from our previous studies have suggested that EA applied at two specific acupoints, Shuigou (GV26) and Neiguan (PC6), may produce significant benefits for cerebral I/R injury, in which the beneficial effects are related to DOR. For example, EA at points Shuigou (GV26) and Neiguan (PC6) could attenuate infarction size and reduce the neurological deficit score of cerebral I/R rats by increasing the expression of DOR, and the effects were specifically blocked by NTI, a DOR antagonist [23]. DOR antagonist NTI can reverse the neuroprotective effect of cumulative EA at Shuigou (GV26) and Neiguan (PC6) on cerebral I/R as well [24]. These results together suggested that DOR activation is involved in the mechanism of the neuroprotection of EA at Shuigou (GV26) and Neiguan (PC6) against cerebral I/R injury. In addition, prior to cerebral I/R injury, cumulative EA treatments could mimic ischemic preconditioning to induce protective function to the brain, presenting reduced infarct volume and improved neuronal function in rats after cerebral ischemia, and this protection is selectively blocked by DOR antagonist NTI [42]. However, whether DOR activation-induced anti-inflammatory response is involved in the underlying mechanism of EA effects in cerebral I/R injury is of great interest to us.

EA at Neiguan (PC6) effectively attenuates the expression of proinflammatory cytokines in cerebral ischemia and improves neurological deficit score [43]. Furthermore, recent reports show that EA at Shuigou (GV26) inhibits inflammatory reactions and induces functional improvement in motor function in CNS injuries, including stroke and spinal cord injury [44, 45]. However, to our knowledge, there was no such scientific research reporting inhibition of inflammation induced by EA at Shuigou (GV26) and Neiguan (PC6) simultaneously at present. In this work, our data indicated that EA at Shuigou (GV26) and Neiguan (PC6) treatment significantly reduced the expression of proinflammatory cytokines IL-1*β* and increased IL-10 after I/R injury. Also, the scores of neurological deficits and the infarct volume after I/R injury were significantly reduced following EA treatment. While NTI injection was combined with EA, the NTI abolished the EA-induced neuroprotective effect in ischemic infarction and neurological deficits and also intervened on the expression of proinflammatory/anti-inflammatory cytokines, suggesting that EA at Shuigou (GV26) and Neiguan (PC6) inhibits inflammatory responses and could be mediated by DOR. In addition, EA at Shuigou (GV26) or Neiguan (PC6) could upregulate the expression of BDNF in the brain of cerebral I/R injured rats and depressed rats, thereby exerting neuroprotective effects [46, 47]. Based on our previous cell experimental work, we conclude that the neuroprotection by EA at Shuigou (GV26) and Neiguan (PC6) are very likely linked to the inhibition of inflammation via the DOR-BDNF/TrkB pathway after I/R injury.

## 5. Conclusion

In summary, our data shows that DOR activation is crucial to neuroprotection against cerebral I/R injury. The DOR-mediated neuroprotection might be related to inflammation suppression through the BDNF/TrkB pathway in OGD/R-induced PC12 cells injury. Moreover, the neuroprotection of EA might depend on curbing the inflammatory response through the DOR-BDNF/TrkB signaling pathway after cerebral I/R injury. The present study suggests that EA at Shuigou (GV26) and Neiguan (PC6) acupoints is a promising therapeutic tool for patients with cerebral I/R injury.

## Figures and Tables

**Table 1:** Primers information for RT-qPCR.

Gene	Forward (5′-3′)	Reverse (5′-3′)
DOR	GGACGCTGGTGGACATCAAT	CGTAGAGAACCGGGTTGAGG
TNF-*α*	GGCTTTCGGAACTCACTGGA	AGGGAGGCCTGAGACATCTT
IL-1*β*	TCAAGCAGAGCACAGACCTG	GAAGACACGGGTTCCATGGT
IL-6	GCAAGAGACTTCCAGCCAGT	CTGGTCTGTTGTGGGTGGTA
IL-4	ACCGAGAACCCCAGACTTGTT	CAGGGTGCTTCGCAAATTTTAC
IL-10	AGGGTTACTTGGGTTGCCAA	TCAGCTTCTCTCCCAGGGAA

## Data Availability

The data used to support the findings of this study are available from the corresponding author upon request.
